# Murine Typhus Mimicking Preeclampsia with Severe Features

**DOI:** 10.1055/a-2977-4190

**Published:** 2026-10-16

**Authors:** Ryan Findlay, Alysha Panjwani, Luis Pacheco

**Affiliations:** 1 Division of Maternal-Fetal MedicineDepartment of Obstetrics and Gynecology 12338 The University of Texas Medical BranchGalvestonTexasUnited States; 2 12338 John Sealy School of Medicine, The University of Texas Medical BranchGalvestonTexasUnited States

**Keywords:** Rickettsia typhi, murine typhus, pregnancy, preeclampsia, HELLP syndrome, thrombocytopenia, headache, severe preeclampsia

## Abstract

**Background** Murine typhus, a flea-borne rickettsiosis endemic to southern Texas, can cause headache, thrombocytopenia, and transaminitis that overlap with the criteria for preeclampsia with severe features.

**Case** A 22-year-old gravida 5 with gestational hypertension presented at 32 ^5/7^ weeks of gestation with 5 days of fever and headache. Worsening thrombocytopenia, transaminitis, and refractory headache were initially attributed to preeclampsia with severe features, and magnesium sulfate was administered. Persistent fever prompted empiric doxycycline for suspected murine typhus; defervescence occurred within 48 hours, and acute serology supported the diagnosis. She delivered a healthy neonate at 37 weeks.

**Conclusion** In endemic regions, murine typhus should be considered in febrile pregnant patients with preeclampsia-like laboratory findings, and empiric doxycycline should not be delayed for serologic confirmation.

## Introduction

Murine typhus is an acute febrile illness caused by *Rickettsia typhi* , an obligate intracellular gram-negative bacterium transmitted to humans by inoculation of infected flea feces into broken skin or mucous membranes. Although the classic reservoir is the rat and its flea *Xenopsylla cheopis* , a suburban transmission cycle involving opossums, cats, and the cat flea *Ctenocephalides felis* sustains disease in the United States, where most cases occur in southern Texas and southern California. 1 Reported cases in Texas have increased over the past two decades, with seasonal peaks in the summer and fall. 1 2

Clinical diagnosis is challenging, as the classic triad of fever, headache, and rash is present in only approximately one-third of patients. 3 In pregnancy, the diagnostic challenge is compounded because the characteristic laboratory abnormalities of murine typhus—elevated liver enzymes and lactate dehydrogenase, thrombocytopenia, hyponatremia, and proteinuria—overlap directly with the criteria for preeclampsia with severe features and HELLP (hemolysis, elevated liver enzymes, and low platelet count) syndrome. 3 4 5 Fewer than 100 pregnancies complicated by scrub or murine typhus have been reported worldwide; among them, poor neonatal outcomes are frequent, and maternal deaths secondary to septic shock have been described. 6 7 We report a case of murine typhus in a patient with gestational hypertension in which the infection was initially interpreted as the development of preeclampsia with severe features, leading to unnecessary interventions.

## Case

A 22-year-old gravida 5 para 2 entered prenatal care in the second trimester. Her antenatal course was notable for gestational hypertension and a self-limited pruritic rash at 26 weeks of gestation that resolved with topical corticosteroids. At 32 ^0/7^ weeks of gestation, she presented to the obstetric triage unit at our institution with new-onset headache and elevated blood pressure readings at home. She was normotensive on evaluation, laboratory assessment for preeclampsia was unremarkable, and her headache resolved with metoclopramide and diphenhydramine. She was discharged with warning precautions.

After 5 days, the patient presented with fever, frontal headache refractory to acetaminophen, cough, nausea, and vomiting. Of note, her young son had a concurrent febrile illness. On triage evaluation, she was febrile to 102.9°F (39.4 °C). Initial laboratory studies demonstrated thrombocytopenia (platelet count: 121 × 10 ^9^ /L), mild transaminitis (aspartate aminotransferase [AST]: 51 U/L, alanine aminotransferase [ALT]: 46 U/L), elevated lactate dehydrogenase (477 U/L), hyponatremia, anemia (hemoglobin: 10.2 g/dL), and a urine protein-to-creatinine ratio of 0.5 ( Table 1 ). Scattered erythematous macules were noted on the left arm and upper back. She was admitted for maternal and fetal monitoring, and a broad evaluation for fever was undertaken, including chest radiography, respiratory viral testing (influenza, SARS-CoV-2, and respiratory syncytial virus), urine culture, blood cultures, and stool studies; all were unremarkable. Empiric amoxicillin and azithromycin were started for possible community-acquired pneumonia given the history of cough.

**Table 1:** Select laboratory values across the clinical course.

Laboratory test	Admission (32 ^5/7^ wk)	Hospital nadir or peak ^a^	48 h after doxycycline	Outpatient follow-up ^b^
Platelets (×10 ^9^ /L)	121	91	192	333
AST (U/L)	51	63	30	19
ALT (U/L)	46	51	39	18
Lactate dehydrogenase (U/L)	477	—	229	157

Abbreviations: ALT, alanine aminotransferase; AST, aspartate aminotransferase; —, not measured at that time point.

Note: Doxycycline was initiated on hospital day 6.

^a^ Worst value recorded during hospitalization.

^b^ At 1–2 weeks after discharge.

On hospital day 3, in the setting of elevated blood pressures recorded more than 4 hours apart superimposed on known gestational hypertension, unrelenting headache unresponsive to analgesia, and worsening thrombocytopenia (nadir: 91 × 10 ^9^ /L), the patient was diagnosed with preeclampsia with severe features. Magnesium sulfate was administered for seizure prophylaxis, together with glucocorticoids to induce fetal lung maturity. Over the next 2 days, the headache resolved and the platelet count improved, and magnesium sulfate was discontinued. Fever, however, persisted despite antibiotic therapy. On hospital day 5, the combination of persistent fever, headache, hyponatremia (nadir sodium: 130 mEq/L), thrombocytopenia, and transaminitis (peak AST: 63 U/L) in a resident of a region endemic for murine typhus prompted suspicion for this diagnosis. Acute *R. typhi* serology was obtained, and empiric doxycycline 100 mg twice daily was initiated on hospital day 6.

The patient defervesced within 48 hours of doxycycline initiation, with rapid improvement in platelet count and transaminases ( Table 1 ). She was discharged on hospital day 8 to complete a 7-day course of doxycycline. Serology against *R. typhi* showed an immunoglobulin M (IgM) titer greater than 1:64 and an immunoglobulin G (IgG) titer less than 1:64, consistent with acute infection. At follow-up 1 week after discharge, the patient was asymptomatic, with a platelet count of 333 × 10 ^9^ /L and normal liver transaminases. The severe features observed during her admission were ultimately attributed to murine typhus rather than to progression of her hypertensive disease, and the diagnosis of preeclampsia with severe features was removed. She underwent medically indicated induction of labor at 37 ^2/7^ weeks of gestation for gestational hypertension and had an uncomplicated spontaneous vaginal delivery of a 3,440-g healthy neonate with Apgar scores of 8 and 9; the newborn was discharged home on day of life 1.

## Comment

This case illustrates the diagnostic overlap between murine typhus and the severe features of preeclampsia. In a patient with an established hypertensive disorder of pregnancy, worsening headache, thrombocytopenia, and transaminitis were reasonably interpreted as disease progression, and management for preeclampsia with severe features—including magnesium sulfate, glucocorticoids, and planned preterm delivery—was initiated in accordance with national guidance. 5 The distinction mattered; had persistent fever not prompted continued infectious evaluation, this patient could have undergone indicated preterm delivery at 34 weeks of gestation for what proved to be a treatable, non-obstetric febrile illness.

The laboratory profiles of the two conditions converge to a striking degree. Murine typhus characteristically produces elevated liver enzymes, elevated lactate dehydrogenase, hypoalbuminemia, thrombocytopenia, and hyponatremia. 2 3 While thrombocytopenia and transaminitis are shared with preeclampsia, new-onset proteinuria—present in this patient—may favor the diagnosis of preeclampsia. During pregnancy, hypoalbuminemia is physiologic and therefore nonspecific, whereas hyponatremia is not an expected feature of hypertensive disease and was a key discriminating finding in this case. Additional features favoring infection included persistent fever refractory to antipyretics, an erythematous macular rash, a symptomatic household contact, cough and gastrointestinal symptoms, and the absence of sustained severe-range blood pressures.

Serologic confirmation by indirect immunofluorescence antibody assay, which is the reference standard, requires a 4-fold rise in IgG titer between acute and convalescent specimens, and titers are frequently negative during the first 7 to 10 days of illness. 8 9 Acute serology therefore cannot exclude the diagnosis, and treatment should not be delayed while awaiting results, as severe maternal disease may ensue. 7 In our patient, an acute IgM titer greater than 1:64 with a negative IgG titer supported recent infection, and defervescence within 48 hours of doxycycline initiation—a well-described therapeutic response—served as pragmatic clinical confirmation. 9 Convalescent titers were planned for her postpartum visit and were pending at the time of this report. Notably, azithromycin has in vitro activity against *R. typhi* and may have contributed to the transient improvement in platelet count observed before doxycycline was begun, but fever persisted until doxycycline was initiated, and clinical evidence supporting azithromycin for typhus in pregnancy is limited. 6

Doxycycline is the treatment of choice for murine typhus in all patients, including pregnant women. 9 Although tetracyclines have historically been avoided in pregnancy, doxycycline differs meaningfully from older agents in this class. A systematic review found no association between doxycycline use and teratogenicity or dental staining in children, and no maternal hepatotoxicity has been reported at commonly used doses. 9 10 The consequences of withholding or delaying effective therapy are not trivial, as longer fever clearance times have been associated with miscarriage, stillbirth, preterm birth, and low birth weight. 6

The present case offers two pivotal teaching points. First, clinicians practicing in regions where murine typhus is endemic, including the Texas Gulf Coast and southern California, should maintain awareness of this flea-borne rickettsiosis and consider it in pregnant patients whose febrile illness and laboratory abnormalities are not fully explained by an obstetric diagnosis. Second, because fever is not a manifestation of the hypertensive disorders of pregnancy, persistent fever should prompt a thorough infectious evaluation even when criteria for preeclampsia with severe features appear to be met.

## Data Availability

All data supporting the findings of this case report are contained within the article. Additional de-identified clinical data are not publicly available to protect patient privacy and confidentiality and may be made available from the corresponding author on reasonable request.

## Declaration of GenAI Use

The authors used a generative artificial intelligence (AI) tool (Claude, Anthropic PBC) during preparation of this manuscript for language editing, formatting, and restructuring of the manuscript to journal specifications. Generative AI was not used for study design, data collection, data analysis, or interpretation of the clinical findings. All clinical content was derived from the medical record and from the cited peer-reviewed literature, which was reviewed by the authors. The authors reviewed and edited all AI-assisted output and take full responsibility for the content, accuracy, and integrity of this manuscript.
